# A Novel Function of CD82/KAI1 in Sialyl Lewis Antigen-Mediated Adhesion of Cancer Cells: Evidence for an Anti-Metastasis Effect by Down-Regulation of Sialyl Lewis Antigens

**DOI:** 10.1371/journal.pone.0124743

**Published:** 2015-04-29

**Authors:** Naoya Yoshihama, Koujiro Yamaguchi, Satomi Chigita, Mariko Mine, Masakazu Abe, Kotaro Ishii, Yosuke Kobayashi, Naonari Akimoto, Yoshihide Mori, Tsuyoshi Sugiura

**Affiliations:** 1 Section of Oral and Maxillofacial Surgery, Division of Maxillofacial Diagnostic and Surgical Sciences, Faculty of Dental Science, Kyushu University, 3-1-1 Maidashi, Higashi-ku, Fukuoka, 812–8582, Japan; 2 Department of Maxillofacial Diagnostic and Surgical Science, Field of Oral and Maxillofacial Rehabilitation, Graduate School of Medical and Dental Science, Kagoshima University, 8-35-1 Sakuragaoka, Kagoshima, 890–8544, Japan; Seoul National University, REPUBLIC OF KOREA

## Abstract

We have recently elucidated a novel function for CD82 in E-cadherin-mediated homocellular adhesion; due to this function, it can inhibit cancer cell dissociation from the primary cancer nest and limit metastasis. However, the effect of CD82 on selectin ligand-mediated heterocellular adhesion has not yet been elucidated. In this study, we focused on the effects of the metastasis suppressor CD82/KAI1 on heterocellular adhesion of cancer cells to the endothelium of blood vessels in order to further elucidate the function of tetraspanins. The over-expression of CD82 in cancer cells led to the inhibition of experimentally induced lung metastases in mice and significantly inhibited the adhesion of these cells to human umbilical vein epithelial cells (HUVECs) *in vitro*. Pre-treatment of the cells with function-perturbing antibodies against sLe^a/x^ significantly inhibited the adhesion of CD82-negative cells to HUVECs. In addition, cells over-expressing CD82 exhibited reduced expression of sLe^a/x^ compared to CD82-negative wild-type cells. Significant down-regulation of ST3 β-galactoside α-2, 3-sialyltransferase 4 (ST3GAL4) was detected by cDNA microarray, real-time PCR, and western blotting analyses. Knockdown of *ST3GAL4* on CD82-negative wild-type cells inhibited expression of sLe^x^ and reduced cell adhesion to HUVECs. We concluded that CD82 decreases sLe^a/x^ expression via the down-regulation of *ST3GAL4* expression and thereby reduces the adhesion of cancer cells to blood vessels, which results in inhibition of metastasis.

## Introduction

Metastasis is a multistep phenomenon characterised by migration of tumour cells from their primary site, invasion of the host blood or lymphatic vessels, seeding of distant organs, and the subsequent development of metastatic tumours. The extravasation of malignant cells involves the interaction of P- and E-selectin, which are cell adhesion molecules found on the surface of endothelial cells that line the blood vessels, with the corresponding carbohydrate ligands occurring on the surface of malignant cells [1]. Several molecular species of carbohydrate ligands for selectins are expressed on cancer cells, including sialyl Lewis X (sLe^x^) and sialyl Lewis A (sLe^a^). Numerous clinical studies have reported that the expression of sLe^x^ and sLe^a^ on tumour cell mucins is directly correlated with metastasis, tumour progression, and poor prognosis [2,3], and it is known that the expression of sLe^x/a^ is markedly enhanced in solid tumours. However, the molecular mechanism underlying the regulation of sLe^x/a^ in cancer cells is not well understood.

Tetraspanins, or TM4SF proteins, comprise a large group transmembrane proteins occurring on the cell surface, which can form complexes with membrane receptors such as integrins. Some tetraspanin-family proteins have been reported to play a particularly important role in tumour cell metastasis [4,5]. CD82/KAI1, a member of the tetraspanin superfamily, was first identified as a T-cell accessory molecule [6] and subsequently identified in a genetic screen for cancer metastasis suppressor genes [7]. In malignant solid tumours, the expression of CD82/KAI1 strongly correlates with a better prognosis for cancer patients, whereas its down-regulation is commonly found in clinically advanced cancers. This data suggest that CD82/KAI1 is a suppressor of invasion and metastasis of various types of solid tumours. [8,9]. Consistent with these observations, it has frequently been observed that expression of CD82 is inversely correlated with the invasive and metastatic potential of cancers of the breast, bladder, colon, cervix, gastrointestinal tract, skin, lung, prostate, pancreas, liver, and thyroid [10–13]. CD82 regulates cell aggregation, cell motility, cancer metastasis, and apoptosis [14]. We have reported that CD82 stabilizes E-cadherin-β-catenin complexes by inhibiting β-catenin tyrosine phosphorylation. This function strengthens the homocellular adhesion of cancer cells and prevents cancer cells from escaping from primary nests [15]. Conversely, once tumour cells invade the blood or lymphatic vessels, heterophilic intercellular adhesion between tumour cells and endothelial cells of the vessels is required as the initial step of metastasis to distant organs. Sialyl Lewis antigens on the cancer cells are involved in adhesion to selectin on endothelial cells of the vessels [16]. However, the effect of CD82 on selectin ligand-mediated cell adhesion has not yet been elucidated.

We here investigated the effects of the metastasis suppressor CD82/KAI1 on the process of heterocellular adhesion of tumour cells to the endothelium of blood vessels, in order to further elucidate the function of tetraspanins. We first demonstrated that sialyl Lewis antigen synthesis is regulated by a CD82/KAI1-mediated system, and then examined the effects of this mechanism on cancer cell metastasis in a mouse metastasis model.

## Materials and Methods

### Antibodies and reagents

Mouse monoclonal antibodies (G-2) and rabbit polyclonal antibodies (C-16) against KAI1 were purchased from Santa Cruz Biotechnology (Santa Cruz, CA, USA). The following function-perturbing antibodies were used: anti- sLe^x^ (mouse, monoclonal) and anti-sLe^a^ (mouse, monoclonal) antibodies, which were obtained from Santa Cruz Biotechnology and MILLIPORE (Temecula, CA, USA), respectively, as well as a mouse monoclonal antibody against β1 integrin, which was obtained from Sigma (St. Louis, MO, USA).

### Cell culture

The human cell line h1299 (a non-small cell lung carcinoma cell line) and its transfectant cell lines, h1299/zeo and h1299/CD82, were established in our laboratory by means of transfection of a control vector or CD82 cDNA, respectively, and a cell sorting-based clone selection technique, as described previously [14]. The cells were grown at 37°C in an atmosphere of 5% CO_2_ in Dulbecco’s modified Eagle’s medium (DMEM; Sigma), supplemented with 10% foetal bovine serum (FBS; ICN Biomedicals, Aurora, OH, USA) and 2 mM L-glutamine.

The two cell lines used in this study, h1299/zeo and h1299/CD82, have been described previously [10]. h1299/zeo is a mock transfected cell line that exhibits weak CD82 expression, whereas h1299/CD82 cells over-express CD82 following cDNA transfection. Immunoblotting analysis showed that the level of CD82 protein in h1299/CD82 cells was 20 times higher than in the wild-type or h1299/zeo cells, whereas flow cytometry revealed that the cell surface level of CD82 in h1299/CD82 cells was approximately 9-fold that of the wild-type or h1299/zeo cells.

### Animals and metastasis assay

Eight-week-old female athymic nude mice (BALBc cAJcl-nu) were purchased from Kyudo (Fukuoka, Japan). The mice were housed in laminar flow cabinets under specific pathogen-free conditions and fed autoclaved water and diets in facilities approved by Kyushu University. Each cabinet contained 1–4 mice for experimental grouping. The animal experimental protocols were approved by the Animal Care and Use Committee of Kyushu University (Permit Number: A23-13-1).

For the experimental lung metastasis studies, 1.0 × 10^6^ cells or vehicle (PBS) only were injected via the tail vein. Mice were randomly separated into the following 4 groups: (A) no treatment (Control: injected with PBS only), (B) h1299, (C) h1299/zeo, and (D) h1299/CD82. Each group contained at least 3 mice (a total of 20 mice were used). Eight weeks after injection, the mice were sacrificed by the administration of pentobarbiturate (100–120 mg/kg) to minimize suffering and the lungs were harvested. Lung metastatic foci were counted if they could be seen with the naked eye as described previously [15].

### Immunoblot analysis

Cell lysates for immunoblotting were prepared in cell lysis buffer (1% Triton X-100, 2 mM sodium orthovanadate, 500 mM NaCl, 10 mM MgCl_2_, 10 μg/mL leupeptin, 10 μg/mL aprotinin, 1 mM PMSF, 50 mM Tris—HCl pH 7.2). The cell lysates were resolved by SDS-PAGE, transferred to a nitrocellulose membrane (Bio-Rad, Hercules, CA, USA), and incubated with specific primary antibodies. Protein bands were visualized using horseradish peroxidase (HRP)-conjugated secondary antibodies and enhanced chemiluminescence reagent (Amersham Pharmacia Biotech, Piscataway, NJ, USA). The bands were scanned by computer-assisted densitometry (ChemiDoc XRS-J; Bio-Rad) and analysed using Quantity One software (Bio-Rad) as described previously [14, 15].

### Fluorescent labelling of live cells and cell adhesion assay

Live h1299 cells were labelled using Vybrant DiO and DiD cell-labelling solutions (Molecular Probes, Eugene, OR, USA) according to the manufacturer’s instructions.

To quantify tumour cell adhesion to human umbilical vein epithelial cells (HUVECs), a standard static adhesion assay was performed, as described previously [17]. HUVECs (1.5 × 10^4^ cells) were placed in 96-well microtiter plates pre-coated with 0.1% gelatin (Sigma) and cultured in low-glucose DMEM with 20% FBS and 0.1 μg/mL basic-FGF for 2–3 days to establish confluent HUVEC monolayers. Fluorescently labeled h1299 cells (4.0 × 10^4^ cells/well) were added to the endothelial monolayer and allowed to adhere at 37°C for 30 min.

To examine the effects of function-perturbing antibodies on adhesion of cancer cells to the HUVEC monolayer, h1299 cells were pre-treated with 5 μg/mL of the function-perturbing antibodies at 37°C for 30 min, and were then applied to the HUVEC monolayer.

The wells were washed 3 times with phosphate-buffered saline (PBS) and cells fixed with methanol for 15 min at room temperature. The adherent cells were quantified under a light microscope using a high power field (× 200). For each of the triplicate experiments, the number of cells in 5 randomly chosen fields was determined and the counts were averaged.

### Transfection of short hairpin RNA (sh RNA)

Cultured h1299/CD82 and h1299/zeo cells were transfected using Lipofectamine (Invitrogen Life Technologies, Carlsbad, CA, USA) according to the manufacturer’s instructions. The h1299/CD82-sh. control and h1299/CD82-sh.CD82 cell lines were generated by transfection of h1299/CD82 cells with the pLKO.1-puro Control Vector (Sigma) and pLKO.1-puro/sh.CD82: NM_002231 (Sigma), respectively [18]. The h1299/zeo-sh.control and h1299/zeo-sh.ST3GAL4 cell lines were generated by Lipofectamine-mediated transfection of h1299/zeo cells with the pLKO.1-puro Control Vector and pLKO.1-puro sh.ST3GAL4:NM_06081105 (Sigma), respectively. Colonies exhibiting resistance to puromycin (Sigma) from the individual transfection experiments were pooled. The expression level of CD82 and ST3GAL4 in shRNA transfected h1299 cells was monitored by RT-PCR and immunoblotting. These cells were maintained in DMEM containing 10% FBS and 2 μg/mL puromycin.

### DNA microarray

Total RNA was extracted from h1299/zeo and h1299/CD82 cells using TRIzol reagent (Invitrogen, Carlsbad, CA, USA). DNA microarray hybridization and scanning were performed by Affymetrix (Santa Clara, CA, USA), using the Affymetrix gene chip HG-U133A plus2.0. GCRMA in Bioconductor (http://www.bioconductor.org) was used for probe analysis and normalization of the microarray data.

Statistical analysis (Student’s *t*-test) and a fold-changes filter (> 3.0) (h1299/CD82 vs. h1299/zeo) were performed sequentially to select the significant genes. Using the gene definition feature, all transferases that regulate sialyl Lewis antigens were identified and are listed in Tables 1 and 2.

**Table 1:** Transferases increased in h1299/CD82 cells (Expression levels of possible regulators of sialyl Lewis antigens analysed by DNA microarray).

Gene Title	Gene Symbol	Fold-Change	Change p-value
Lysophosphatidylglycerol acyltransferase1	*LPGAT1*	1.74110113	0.000618
UDP-GlcNAc:betaGal beta-1,3-N-acetylglucosaminyltransferase-like 1	*B3GNTL1*	1.51571657	0.000241
Rab geranylgeranyltransferase, beta subunit	*RABGGTB*	1.41421356	0.000035
dihydrolipoamide S-acetyltransferase	*DLAT*	1.31950791	0.000438
(E2 component of pyruvate dehydrogenase complex)			
MAK10 homolog, amino-acid N-acetyltransferase subunit, (S. cerevisiae)	*MAK10*	1.31950791	0.001336
Glycosyltransferase 28 domain containing 1	*GLT28D1*	1.23114441	0.000389
Aminoadipate-semialdehyde dehydrogenase-phosphopantetheinyl transferase	*AASDHPPT*	1.23114441	0.000088
Dihydrolipoamide S-acetyltransferase	*DLAT*	1.23114441	0.001336
(E2 component of pyruvate dehydrogenase complex)			
Phosphoribosyl transferase domain containing 1	*PRTFDC1*	1.23114441	0.000492
Diacylglycerol O-acyltransferase homolog 2 (mouse)	*DGAT2*	1.23114441	0.000692
Glutathione S-transferase kappa 1	*GSTK1*	1.14869835	0.001077

**Table 2:** Transferases reduced in h1299/CD82 cells (Expression levels of possible regulators of sialyl Lewis antigens analysed by DNA microarray).

Gene Title	Gene Symbol	Fold-Change	Change p-value
ST3 beta-galactoside alpha-2,3-sialyltransferase 4	*ST3GAL4*	0.05440941	0.999911
Protein arginine methyltransferase 2	*PRMT2*	0.116629124	0.998799
Monoacylglycerol O-acyltransferase 2	*MOGAT2*	0.153893052	0.999135
Heparan sulfate (glucosamine) 3-O-sulfotransferase 6	*HS3ST6*	0.189464571	0.998349
Monoacylglycerol O-acyltransferase 1	*MOGAT1*	0.251482635	0.998799
Farnesyl diphosphate synthase(farnesyl pyrophosphate synthetase, dimethylallyltranstransferase, geranyltranstransferase)	*FDPS*	0.466516496	0.999226
UDP-N-acetyl-alpha-D-galactosamine:polypeptide N-acetylgalactosaminyltransferase 6 (GalNAc-T6)	*GALNT6*	0.535886731	0.999786
Carbohydrate (chondroitin 4) sulfotransferase 11	*CHST11*	0.615572207	0.998514
UDP-Gal:betaGal beta 1,3-galactosyltransferase polypeptide 6	*B3GALT6*	0.707106781	0.999654
tRNA 5-methylaminomethyl-2-thiouridylate methyltransferase	*TRMU*	0.707106781	0.998923
ST6 (alpha-N-acetyl-neuraminyl-2,3-beta-galactosyl-1,3)-N-acetylgalactosaminide alpha-2,6-sialyltransferase 4	*ST6GALNAC4*	0.757858283	0.999833
UDP-GlcNAc:betaGal beta-1,3-N-acetylglucosaminyltransferase 5	*B3GNT5*	0.757858283	0.99998
1-acylglycerol-3-phosphate O-acyltransferase 2 (lysophosphatidic acid acyltransferase, beta)	*AGPAT2*	0.757858283	0.999689
Farnesyl diphosphate synthase (farnesyl pyrophosphate synthetase, dimethylallyltranstransferase, geranyltranstransferase)	*FDPS*	0.812252396	0.999811
Catechol-O-methyltransferase	*COMT*	0.812252396	0.999448
Farnesyl-diphosphate farnesyltransferase 1	*FDFT1*	0.812252396	0.99997
Transcribed locus, strongly similar to NP_001488.2 UDP-Gal:betaGlcNAc beta 1,4- galactosyltransferase 1, membrane-bound form; lactose synthase A protein; glycoprotein-4-beta-galactosyltransferase 2 [Homo sapiens]	*---*	0.812252396	0.999382

### Real-time reverse transcriptase (RT)-polymerase chain reaction (PCR)

Total RNA was extracted from h1299 cells using TRIzol (Invitrogen, Carlsbad, CA, USA) and used for first-strand cDNA synthesis. The mRNA levels were quantified in triplicate using a real-time PCR system with the Brilliant SYBR Green qPCR Kit (Stratagene, La Jolla, CA, USA) as described previously [18]. The specific primers for glycosyltransferase were as follows:(*ST3GAL1*: 5′-GCATAACGCCCATATAGAT-3′ and 5′-AAGGCTCTGACTGCTCTGT-3′; *ST3GAL2*: 5′-CTGGATGCTGGGACCTAC-3′ and 5′-GCTACTTGGAAGGCTGAGG-3′; *ST3GAL3*: 5′-TCCTGGACGCACAATATC-3′ and 5′-GGTCTGAAGACTCCTGTGTA-3′; *ST3GAL4*: 5′-AGTAGAAAACAACCCAGACAC-3′ and 5′-AGAGGTTGAGAATCCGAA-3′; and *ST3GAL5*: 5′-TGCCTCAGTTCACCCTCA-3′ and 5′-TGGTGGTGTTTGTGTGCTG-3′.

The PCR cycling conditions were 10 min at 95°C for 1 cycle followed by 45 cycles each of 95°C for 30 s, 60°C for 30 s, and 72°C for 60 s. Amplicons are confirmed that signals are unique by dissociation curve analyses. Expression levels for each sample, obtained from parallel assays, were normalized to those of the gene encoding glyceraldehyde-3-phosphate dehydrogenase (*GAPDH*) and were analysed using the LightCycler2.0 System software package (Roche Applied Science, Indianapolis, IN, USA).

## Results

### Ectopic expression of CD82 inhibits lung metastasis in mice

**Fig 1:**
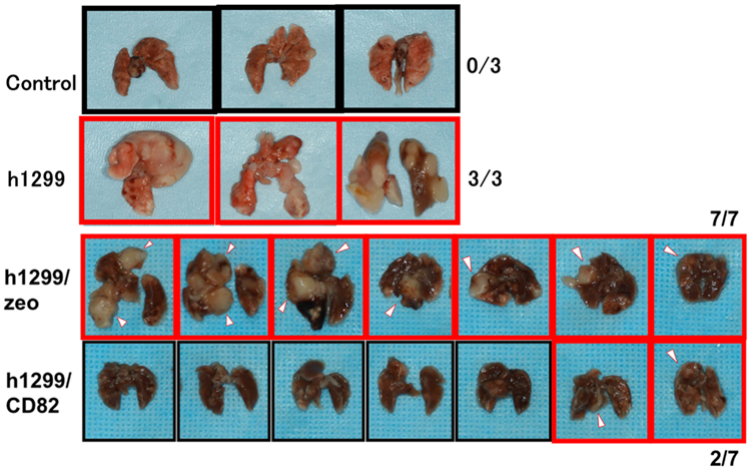
Inhibitory effect of CD82 on cancer cell metastasis in a mouse metastasis model. The tail veins of nude mice were injected with 1.0 × 10^6^ h1299/zeo or h1299/CD82 cells. Eight weeks after injection, the mice were killed and the lungs recovered. Metastatic foci were counted by eye. The injection of h1299/zeo cells resulted in 100% lung metastasis (7/7), whereas h1299/CD82 cells showed a significantly lower rate of metastasis (28.5%; 2/7).

In the first experiment, we confirmed the metastasis-inhibitory effect of CD82 that has previously been reported using an animal metastasis model [17]. For the experimental lung metastasis studies, we injected h1299 cells into the tail vein of 8-week-old female athymic nude mice. Eight weeks after injection, any metastatic foci were observed and counted visually. CD82 showed a strong inhibitory effect on the size and number of lung metastasis foci (Fig 1). Specifically, mice transfected with h1299/zeo cells showed 100% lung metastasis (7/7), whereas the CD82 transfectants, h1299/CD82, exhibited a significant inhibition of metastasis, with a metastasis rate of only 28.5% (2/7). Given that the lung metastasis rate directly reflects the extent of cellular adhesion to the lung blood vessels, this result suggested that CD82 plays an important role in cellular adhesion to blood vessels.

### Ectopic expression of CD82 inhibits tumour cell adhesion to HUVECs via sialyl Lewis antigens

**Fig 2:**
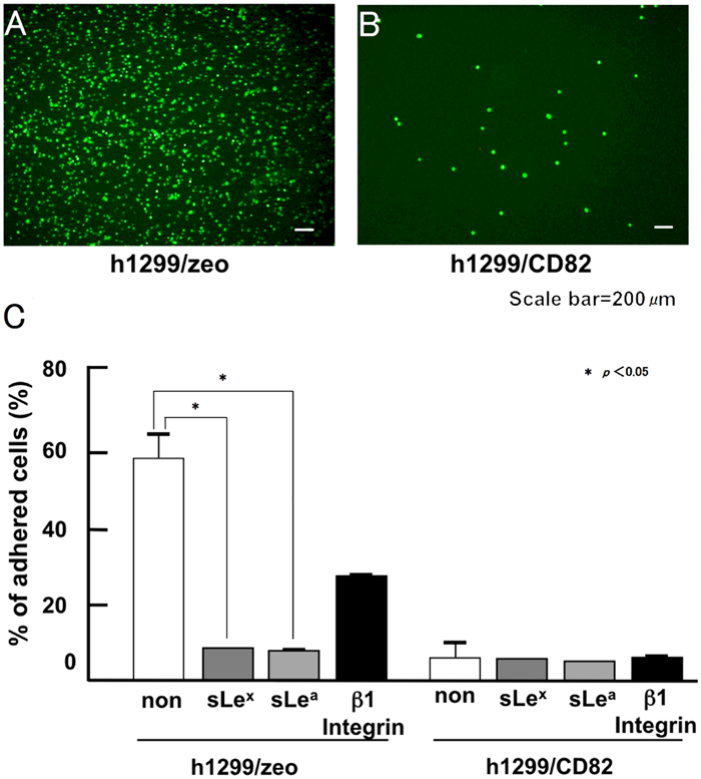
Inhibitory effect of CD82 on cancer cell adhesion to a HUVEC monolayer culture. Fluorescently labeled h1299/zeo or h1299/CD82 cells (4.0 × 10^4^ cells/well) were applied to a HUVEC monolayer culture and allowed to adhere at 37°C. Adhered cells were quantified after 30 min. Adhered h1299/zeo (A) and h1299/CD82 (B) cells and the effect of function-perturbing antibodies on cell adhesion to the HUVEC monolayer were analysed (C). h1299 cells were pre-treated with 5 μg/ml of the indicated antibodies and then applied to the HUVEC monolayer. Experiments were performed in triplicate and the number of adhered cells was averaged. Bars indicate the standard deviation.

The adhesion of cancer cells to blood vessels involves the interaction of P- and E-selectin on the epithelial cells of blood vessels with the corresponding sLe^x^ and sLe^a^ carbohydrate ligands on the surface of cancer cells [12]. We analysed the effect of CD82 on cell adhesion to blood vessels using a previously described cell adhesion assay in HUVECs [11]. It was previously confirmed that HUVECs express both P- and E-selectin [11]. We observed that approximately 65% of the loaded h1299/zeo cells adhered to the HUVEC monolayer. Conversely, h1299/CD82 cells showed significantly less cell adhesion, with only 6.6% adherence to the HUVEC monolayer (Fig 2A and 2B). Pre-treatment with function-perturbing antibodies against sLe^x^, sLe^a^, or β1 integrins inhibited h1299/zeo adhesion to HUVECs by 9.4%, 8.4%, and 30.2%, respectively (Fig 2C). In contrast, function-perturbing antibodies against sLe^x/a^ showed no significant inhibition of h1299/CD82 cell adhesion to HUVECs. These results strongly suggested an inhibitory role for CD82 in sialyl Lewis-mediated cellular adhesion.

### Ectopic expression of CD82 reduces sialyl Lewis synthesis

**Fig 3:**
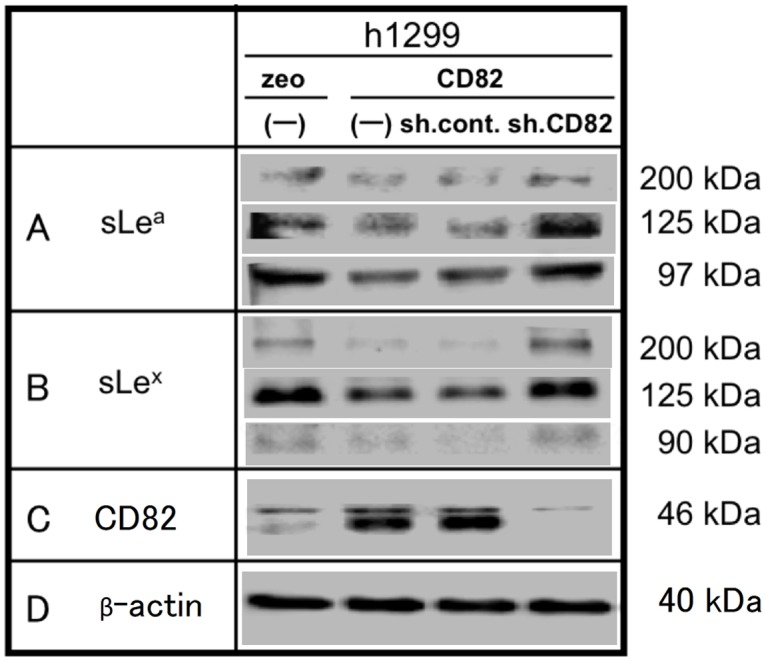
Effects of CD82 on the expression of sialyl Lewis antigens. Whole cell lysates (200 μg) of h1299 cells (h1299/zeo, zeo; h1299/CD82, CD82; h1299/CD82-sh.control, sh.cont; h1299/CD82-sh.CD82, sh.CD82) were resolved by 7.5% SDS-PAGE and analysed by immunoblotting with anti-sLe^a^ (A) or anti-sLe^x^ (B) antibodies. The same blots were stripped and re-probed for β-actin as a loading control. Experiments were repeated in triplicate, and the most representative data are shown.

The expression of sialyl Lewis antigens on h1299 cells was analysed by immunoblotting (Fig 3). sLe^a^ was detected as bands corresponding to approximately 97, 125 and 200 kDa, and its expression level was 5–8-fold higher in h1299/zeo cells than in h1299/CD82 cells (Fig 3A). Similarly, the expression of sLe^x^ proteins was detected at approximately 90, 125, and 200 kDa, and was 5–8-fold higher in h1299/zeo cells than in h1299/CD82 cells (Fig 3B). These findings were in accordance with the results of the HUVEC adhesion assay.

Furthermore, we transfected h1299/CD82 cells with CD82 shRNA to confirm whether this down-regulation of sialyl Lewis antigens is a direct effect of the ectopic expression of CD82. *CD82* shRNA completely inhibited CD82 expression (Fig 3C) and, simultaneously, the production of sLe^a^ and sLe^x^ recovered to the expression levels observed in h1299/zeo cells.

### Ectopic expression of CD82 reduces the mRNA levels of glycosyltransferase genes related to sialyl Lewis synthesis

To examine the mechanisms for the down-regulation of sialyl Lewis antigens by CD82, we performed a global DNA microarray analysis of the possible regulators of sialyl Lewis antigens (all transferases). As shown in Table 1, CD82 markedly down-regulated (0.05-fold change) the expression of *ST3GAL4* (underlined in Table 1). In contrast, the expression of genes encoding other groups of glycosyltransferases and sugar transporter groups was not markedly changed.

**Fig 4:**
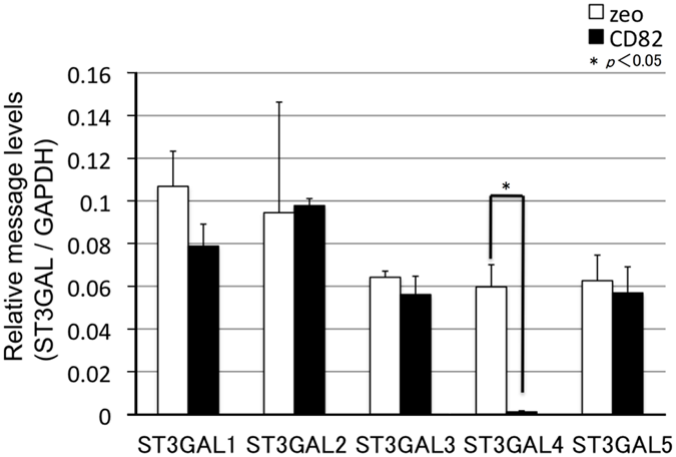
Effects of CD82 on the expression of genes encoding glycosyltransferases (ST3GALs) related to sialyl Lewis synthesis. Total RNA was isolated from h1299 cells and analysed by real-time RT-PCR. mRNA levels of glycosyltransferase-encoding genes *(ST3GALs)* were corrected relative to the levels of *GAPDH* mRNA, with h1299/zeo set at 1. Data are shown as the mean ± SEM.

**Fig 5:**
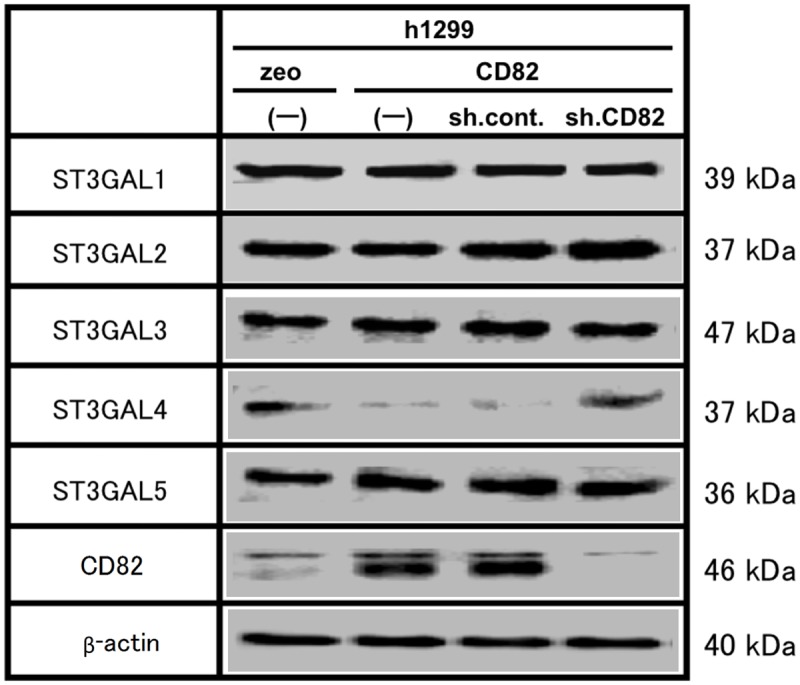
Effects of CD82 on the protein expression of glycosyltransferases related to sialyl Lewis synthesis. Whole cell lysates (300 μg) of h1299 cells (h1299/zeo, zeo; h1299/CD82, CD82; h1299/CD82-sh.control, sh.cont; h1299/CD82-sh.CD82, sh.CD82) were resolved by 10% SDS-PAGE and analysed by immunoblotting with anti-ST3GAL primary antibodies. The same blots were stripped and re-probed for β-actin and CD82. Experiments were repeated in triplicate, and the most representative data are shown.

We next examined the effects of CD82 on the mRNA and protein levels of ST3GALs using real time PCR (Fig 4) and immunoblotting (Fig 5), respectively. The ectopic expression of CD82 had no effect on the expression of ST3GALs, except for a marked decrease in the *ST3GAL4* mRNA and protein levels.

We also assessed whether the reduction of *ST3GAL4* in h1299/CD82 cells was a CD82-specific event by using CD82 knockdown. Following knockdown of CD82, the protein level of ST3GAL4 completely recovered to the level observed in h1299/zeo cells.

### *ST3GAL4* shRNA reduces sLex production

**Fig 6:**
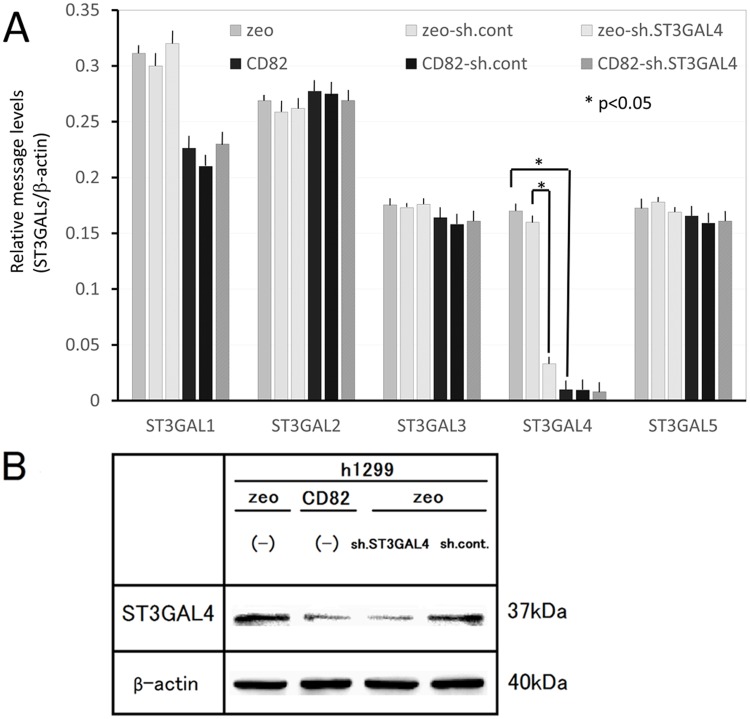
Effect of *ST3GAL4* knockdown on mRNA levels and protein levels. A. Total RNA was isolated from h1299 cells and analysed by real-time RT-PCR. mRNA levels of *ST3GALs* were corrected relative to the levels of β-actin mRNA, with those of h1299/zeo set at 1. Data are shown as the mean ± SEM. B. Whole cell lysates (300 μg) of h1299 cells (h1299/zeo, zeo; h1299/CD82, CD82; h1299/zeo-sh.ST3GAL4, sh.ST3GAL4; h1299/zeo-sh.control, sh.cont) were resolved by 10% SDS-PAGE and analysed by immunoblotting with anti-ST3GAL4 primary antibodies. The same blots were stripped and re-probed for β-actin. Experiments were repeated in triplicate, and the most representative data are shown.

**Fig 7:**
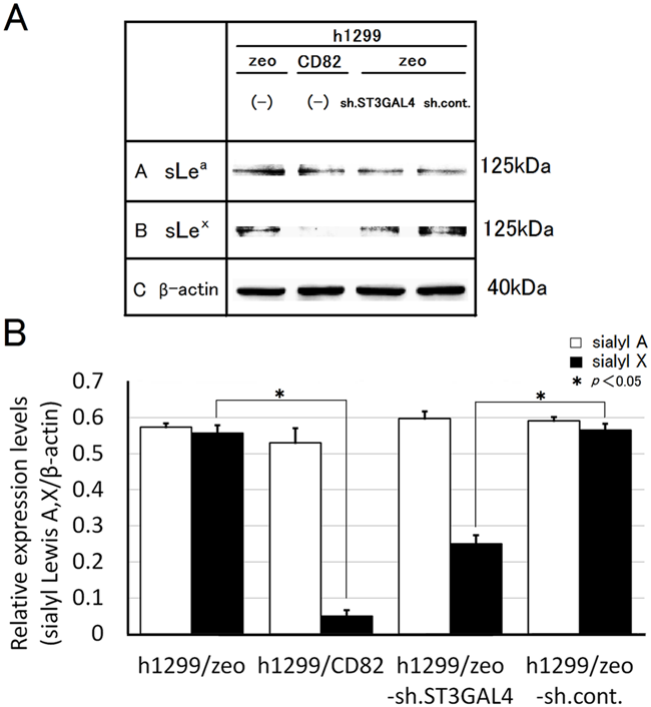
Effects of *ST3GAL4* knockdown on the expression of sialyl Lewis antigens. (A) Whole cell lysates (300 μg) of h1299 cells (h1299/zeo, zeo; h1299/CD82, CD82; h1299/zeo-sh.ST3GAL4, sh.ST3GAL4; h1299/zeo-sh.control, sh.cont) were resolved by 10% SDS-PAGE and analysed by immunoblotting with anti-sLe^a^ or anti-sLe^x^ antibodies. The same blots were stripped and re-probed for β-actin as a loading control. Experiments were repeated 3 times, and the most representative data are shown. (B) Densitometric analysis was performed on (A), followed by normalisation to the densitometric value of β-actin and indicated as “Relative expression value (β-catenin/β-actin)”. Relative expression values from 3 individual experiments were averaged. Bars indicate the standard deviation.

To examine whether down-regulation of ST3GAL4 reduces the production of sLex, we knocked down *ST3GAL4* by stable transfection of *ST3GAL4* shRNA. The expression of *ST3GAL4* was specifically reduced by shRNA at both mRNA and protein levels (Fig 6A and 6B). The protein level of sLe^x^ was significantly reduced in h1299/zeo shST3GAL4 cells (0.3-fold of h1299/zeo sh.cont). In contrast, the protein level of sLe^a^ showed no significant difference between h1299/zeo-sh.cont cells and h1299/zeo-sh.ST3GAL4 cells. The finding that ST3GAL4 specifically reduces sLe^x^ protein production suggested that sLe^x^ production was down-regulated by CD82 expression via *ST3GAL4* down-regulation (Fig 7A and 7B).

### *ST3GAL4* shRNA inhibits cell adhesion to HUVECs

**Fig 8:**
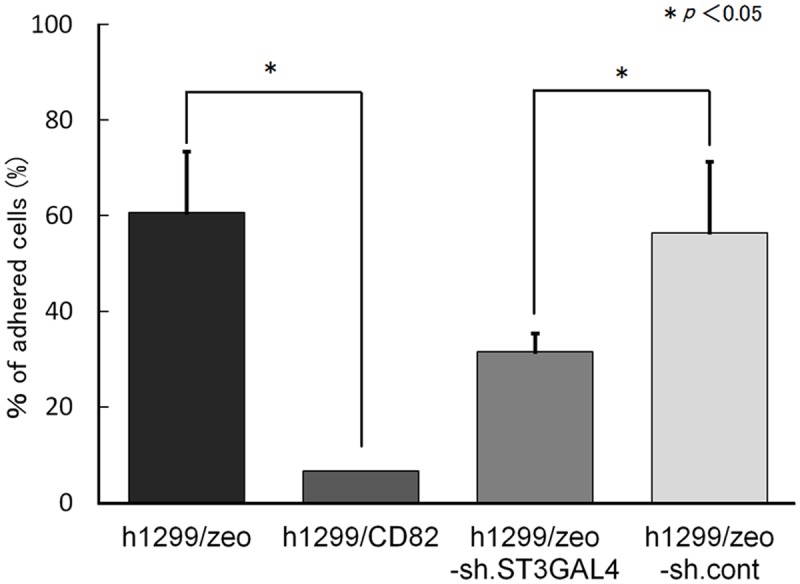
Effects of ST3GAL4 knockdown on cancer cell adhesion to a HUVEC monolayer culture. Fluorescently labelled h1299/zeo, h1299/CD82, h1299/zeo-sh.ST3GAL4, and h1299/zeo-sh.control cells (4.0 × 10^4^ cells/well) were applied to a HUVEC monolayer culture and allowed to adhere at 37°C. Adhered cells were quantified after 30 min. h1299 cells adhered to the HUVEC monolayer were analysed. Experiments were performed in triplicate and the number of adhered cells was averaged. Bars indicate the standard deviation.

Finally, we examined the effect of shRNA-mediated down-regulation of *ST3GAL4* on cell adhesion to HUVECs. The cell adhesion to HUVECs showed approximately 50% inhibition of wild-type h1299/zeo cells after *ST3GAL4* knockdown. However, the inhibition level of h1299/zeo shST3GAL4 cells did not reach that of h1299/CD82 (90% inhibition of h1299/zeo; Fig 8).

## Discussion

CD82/KAI1 has been reported to have anti-metastatic properties and has previously been confirmed to be a suppressor of metastasis. We previously demonstrated a novel function of CD82 in E-cadherin-mediated homophilic intercellular adhesion [15]. In this study, we examined the effects of CD82 on heterophilic cellular adhesion to the blood or lymphatic vessels, which is the initial step of metastasis to distant organs; and demonstrated the novel regulatory role of CD82 in sialyl Lewis-dependent cellular adhesion. In our *in vivo* metastasis assay, CD82 showed a strong inhibitory effect on the size and number of lung metastasis foci (Fig 1) following the direct injection of cancer cells into the mouse tail vein. Thus, our results strongly suggest an inhibitory role for CD82 in the adhesion of cancer cells to vascular endothelial cells.

Sialyl Lewis antigens are involved in the initial step of cancer cell adhesion to blood vessels [16]. Numerous clinical studies have reported that the expression of sLe^x^ and sLe^a^ on tumour cell mucins correlates directly with metastasis, tumour progression, and poor prognosis [2,3]. Once the initial adhesion via sialyl Lewis antigens is established, integrin-mediated adhesion ensues [19]. We demonstrated that addition of an anti-integrin antibody only partially inhibited the adhesion of cancer cells to HUVECs, suggesting that the initial sialyl Lewis antigen-mediated adhesion is essential for cancer cell adhesion to blood vessels (Fig 2). The ectopic expression of CD82 down-regulated the synthesis of sLe^a/x^ (Fig 3), supporting the results of the HUVEC adhesion assay.

The biosynthesis pathway of sLe^a^ and sLe^x^ depends on a variety of enzymes, which catalyse the transfer of sialic acid and fucose to the oligosaccharide side-chains of glycoconjugates. Briefly, *N*-acetyllactosamine (GlcNac) β1 oligosaccharide is synthesized by GlcNAc transferases. Based on GlcNac β1 oligosaccharide substrates, β1, 3-galactosyltransferases (β3Gal-Ts) synthesize α1→3 disaccharides (type 1 chains) and β1, 4-galactosyltransferases (β4Gal-Ts) synthesize α1→4 disaccharides (type 2 chains). Sialyltransferase (ST) belonging to the *ST3GAL* family contains 6 subfamilies of enzymes. Among these, ST3Gal3 sialylates type 1 chains to produce sLe^a^, whereas ST3Gal4 and -6 are required to sialylate type 2 chains for sLe^x^ production.

Sequential addition of α1, 4-linked fucose to type 1 chains and α1, 3-linked fucose to type 2 chains by fucosyltransferase (FUTs) finalizes the biosynthesis of sLe^a^ and sLe^x^, respectively. The expression of sLe^a^ and sLe^x^ is associated with carcinogenesis and tumour progression. The expression of the *ST* and *FUT* genes that encodes the enzymes necessary for the biosynthesis of these antigens is also correlated with these malignant characters. Increased mRNA levels of *ST* and *FUT* are found in several types of malignant tumours. *ST3GAL3* expression in patients with breast cancer was strongly associated with poor prognosis and reduced overall survival [20]. Similarly, the increased expression of *ST3GAL4* and *FUT4* was reported in colorectal carcinomas [21]. Conversely, the down-regulation of *ST3GAL4* has been observed in colorectal cancer tissues and human renal cell carcinoma [22,23]. In gastric cancer, the expression levels of *ST3GAL3* and *FUT4* mRNA were significantly enhanced in carcinoma tissues [24]. Furthermore, the expression of *FUT4* and *FUT7* mRNA was related to poor prognosis in lung cancer patients [25], and the increased activity of α1,3/4-FUTs were observed in ovarian carcinoma compared to healthy tissue [26]Chandrasekaran et al., 1992 E.V. Chandrasekaran, R.K. Jain and K.L. Matta, Ovarian cancer alpha 1,3-L-fucosyltransferase. Differentiation of distinct catalytic species with the unique substrate, 3′-sulfo-N-acetyllactosamine in conjunction with other synthetic acceptors, *J Biol Chem* **267** (1992), pp. 23806–23814. View Record in Scopus Cited By in Scopus (26). These reports suggest that the roles of *ST* and *FUT* differ in various cancer types *in vivo*.

In our model, CD82 significantly down-regulated (0.05-fold change) the expression of *ST3GAL4*. In contrast, the expression of other groups of glycosyltransferases and sugar transporter groups was not markedly changed. Knockdown of *CD82* mRNA by shRNA in h1299/CD82 cells resulted in the recovery of *ST3GAL4* expression and the synthesis of sLe^a/x^, suggesting that CD82 has specific effects on *ST3GAL4* expression.

As mentioned above, *ST3GAL4* usually synthesizes only sLe^x^ and our *ST3GAL4* knockdown study showed results indicating a specific down-regulation of sLe^x^ in h1299/zeo shST3GAL4 cells. In contrast, ectopic expression of CD82 reduced the synthesis of sLe^a^ and sLe^x^, simultaneously. This type of discrepancy has been reported previously. The enzymatic activity of ST3GAL4 recombinant protein was effective for both type 1 and type 2 disaccharides *in vitro*, whereas *in vivo*, it was only effective for type 2 substrates [27]Sasaki et al., 1993 K. Sasaki, E. Watanabe, K. Kawashima, S. Sekine, T. Dohi and M. Oshima *et al*., Expression cloning of a novel Gal beta (1-3/1-4) GlcNAc alpha 2,3-sialyltransferase using lectin resistance selection, *J Biol Chem* **268** (1993), pp. 22782–22787. View Record in Scopus | Cited By in Scopus (113). The data produced in our microarray analysis may provide another mechanism to explain this discrepancy. A weak down-regulation of β*3galT* (0.7-fold change, underlined in Table 1), which synthesizes type 1 disaccharides, was found. It is possible that even this weak down-regulation of β*3galT* can result in a significant down-regulation of sLe^a^, because a type 1 disaccharide is the essential core element of its derivatives (Le^b^, Le^a^, and sLe^a^).

## Conclusions

Together, our data suggest that the ectopic expression of CD82 significantly reduces the levels of sLe^x/a^, which in turn reduces the adhesion of cancer cells to blood vessels. Thus, we propose that the decreased expression of *ST3GAL4* mRNA plays a key role in the CD82-mediated down-regulation of sLe^x/a^, especially that of sLe^x^. Therefore, we conclude that CD82 down-regulates ST3GAL4 and regulates sialyl Lewis antigen-mediated cancer cell adhesion to blood vessels and, thereby, the inhibition of cancer cell metastasis. Our results demonstrate a novel function for CD82 in the regulation of sialyl Lewis antigens.
